# Serial endometrial thickness and risk of non‐endometrial hormone‐dependent cancers in postmenopausal women in UK Collaborative Trial of Ovarian Cancer Screening

**DOI:** 10.1002/uog.21894

**Published:** 2020-08-01

**Authors:** M. Burnell, A. Gentry‐Maharaj, C. Glazer, C. Karpinskyj, A. Ryan, S. Apostolidou, J. Kalsi, M. Parmar, S. Campbell, I. Jacobs, U. Menon

**Affiliations:** ^1^ MRC CTU, Institute of Clinical Trials and Methodology University College London London UK; ^2^ Department of Occupational and Environmental Medicine Frederiksberg‐Bispebjerg University Hospital Copenhagen NV Denmark; ^3^ Department of Women's Cancer, Institute for Women's Health University College London London UK; ^4^ Create Fertility London UK; ^5^ University of New South Wales Sydney Australia

**Keywords:** breast cancer, cancer biomarker, cumulative estrogen, endometrial thickness, joint models, lung cancer, ovarian cancer, transvaginal ultrasound

## Abstract

**Objective:**

Estrogen is a well‐established risk factor for various cancers. It causes endometrial proliferation, which is assessed routinely as endometrial thickness (ET) using transvaginal ultrasound (TVS). Only one previous study, restricted to endometrial and breast cancer, has considered ET and the risk of non‐endometrial cancer. The aim of this study was to explore the association between baseline and serial ET measurements and nine non‐endometrial hormone‐sensitive cancers, in postmenopausal women, using contemporary statistical methodology that attempts to minimize the biases typical of endogenous serial data.

**Methods:**

This was a cohort study nested within the UK Collaborative Trial of Ovarian Cancer Screening (UKCTOCS). In the ultrasound arm of UKCTOCS, 50639 postmenopausal women, aged 50–74, underwent annual TVS examination, of whom 38 105 had a valid ET measurement, no prior hysterectomy and complete covariate data, and were included in this study. All women were followed up through linkage to national cancer registries. The effect of ET on the risk of six estrogen‐dependent cancers (breast, ovarian, colorectal, bladder, lung and pancreatic) was assessed using joint models for longitudinal biomarker and time‐to‐event data, and Cox models were used to assess the association between baseline ET measurement and these six cancers in addition to liver cancer, gastric cancer and non‐Hodgkin's lymphoma (NHL). All models were adjusted for current hormone‐replacement therapy (HRT) use, body mass index, age at last menstrual period, parity and oral contraceptive pill use.

**Results:**

The 38 105 included women had a combined total of 267 567 (median, 8; interquartile range, 5–9) valid ET measurements. During a combined total of 407 838 (median, 10.9) years of follow‐up, 1398 breast, 351 endometrial, 381 lung, 495 colorectal, 222 ovarian, 94 pancreatic, 79 bladder, 62 gastric, 38 liver cancers and 52 NHLs were registered. Using joint models, a doubling of ET increased significantly the risk of breast (hazard ratio (HR), 1.21; 95% CI, 1.09–1.36; *P* = 0.001), ovarian (HR, 1.39; 95% CI, 1.06–1.82; *P* = 0.018) and lung (HR, 1.25; 95% CI, 1.02–1.54; *P* = 0.036) cancers. There were no statistically significant associations between ET and the remaining six cancers.

**Conclusion:**

Postmenopausal women with high/increasing ET on TVS are at increased risk of breast, ovarian and lung cancer. It is important that clinicians are aware of these risks, as TVS is a common investigation. © 2019 The Authors. *Ultrasound in Obstetrics & Gynecology* published by John Wiley & Sons Ltd on behalf of the International Society of Ultrasound in Obstetrics and Gynecology.

**CONTRIBUTION**

**What are the novel findings of this work?**

This is the first study to investigate the association between endometrial thickness (ET) and the risk of eight non‐endometrial hormone‐dependent cancers in postmenopausal women, and has much greater power *vis‐à‐vis* the only prior study that considered breast cancer. A doubling of ET was found to increase significantly the risk of ovarian and lung cancer in addition to breast cancer.

**What are the clinical implications of this work?**

Clinicians performing transvaginal ultrasonography should be aware that postmenopausal women with high/increasing ET are at an increased risk of breast, ovarian and lung cancer.

## INTRODUCTION

Cumulative estrogen exposure is linked to many types of cancers, particularly female cancers^1^. In breast^2^, ^3^, endometrial^4^, ^5^ and, to a lesser extent, ovarian^6^, ^7^, ^8^ cancer, this increase in risk associated with higher levels of estrogen is particularly well established. For lung cancer, there is a growing consensus that estrogens contribute to the increased rates seen in women compared with in men, and that smoking further augments the effect of estrogen^9^, ^10^, ^11^, ^12^. For colorectal cancer, the evidence is unclear^13^, ^14^, with an increased risk reported for estrone^15^ and a decreased risk for estrogen^16^, ^17^. The association between estrogen exposure and risk of gastric^18^, pancreatic^19^ and bladder cancer^20^ is less clear, but there is some evidence of a decreased risk associated with estrogen. For non‐Hodgkin's Lymphoma (NHL), definitive associations with estrogen have not been established^19^, ^21^.

Estrogen receptors are expressed in many tissues, including the endometrium. Cumulative estrogen exposure, especially in postmenopausal women, leads to endometrial proliferation which could be easily assessed as endometrial thickness (ET) using transvaginal ultrasound (TVS). In postmenopausal women, the adipose tissue provides an additional source of estrogen, which in turn causes proliferation of the endometrium^22^. Whilst increased ET is well‐established as an early detection marker for endometrial cancer^23^, ET could also be a potential risk marker for other hormone‐sensitive cancers. A prospective study within the Prostate, Lung, Colorectal and Ovarian (PLCO) screening trial reported that an ET > 5 mm, compared with an ET < 3 mm, was associated with a 2‐fold increase in the risk of breast cancer^24^. This study was limited by sample size (1272 women with 91 breast cancers) and the main analysis was restricted to only the baseline measurement. Furthermore, the statistical method used to incorporate the serial measurements was susceptible to bias.

Using a prospective cohort design in the ultrasound arm of the United Kingdom Collaborative Trial of Ovarian Cancer Screening (UKCTOCS)^25^ and a joint model of longitudinal and time‐to‐event data, the aim of this study was to explore whether ET measurement at baseline, or serial change over 11 years of screening, is associated with the risk of nine estrogen‐dependent non‐endometrial cancers in postmenopausal women.

## METHODS

### Participants

UKCTOCS (ISRCTN22488978; ClinicalTrials.gov NTC00058032) is a multicenter randomized controlled trial of 202 638 postmenopausal women aged 50–74 years, from England, Wales and Northern Ireland, randomized to annual screening either using TVS (ultrasound arm; *n* = 50 639) or serum CA125 (multimodal arm; *n* = 50 640) or to no screening (control arm; *n* = 101 359)^25^. The eligibility criteria and trial details are described elsewhere^25^, ^26^. UKCTOCS was approved by the UK North West MREC (00/8/34) on 23^rd^ June 2000. Participants provided written consent for use of their data in secondary studies. This analysis used data from the ultrasound arm, in which women were offered annual TVS screening between 17^th^ April 2001 and 31^st^ December 2011. We limited the analysis to those who had not undergone hysterectomy prior to recruitment, had complete covariate data and had at least one valid ET measurement.

### TVS data

Women underwent annual TVS scans, with repeat scans performed when abnormal adnexal morphology was noted^25^. Between 2001 and 2008, the scans were performed using a Kretz/Medison SA9900 machine (Medison, Seoul, South Korea), and from 2008 they were performed using a Medison Accuvix XQ machine (Medison). In addition to ovarian morphology, ET measurements were collected routinely and entered in a central web‐based trial management system^26^. ET was recorded at the thickest anteroposterior diameter of the endometrium in a sagittal plane of the uterus. Calipers were placed perpendicular to the outer edge of the endometrium. If there was fluid in the endometrial cavity, the cavity fluid and the double endometrial stripe were measured, and the fluid diameter at the same point was subtracted^23^.

### Cancer notification

All women were flagged with the NHS Digital, for England and Wales and Northern Ireland, Cancer Registry and cancer notifications (site, morphology and date of diagnosis) included in this analysis were diagnosed by 31^st^ December 2014. Nine types of cancer that have a possible association with estrogen were identified using cancer registry ICD‐10 codes: ovarian (C56, C57, C48), breast (C50), lung (C34), colorectal and anal (C18–C21), pancreatic (C25), bladder (C67), liver (C22), gastric (C16) and NHL (C85).

### Covariate data

Data on hysterectomy, height, weight, current or past use of the oral contraceptive pill (OCP), parity, current use of hormone‐replacement therapy (HRT) and age at last menstrual period (LMP) were captured at recruitment^25^. Approximately 77% of women also completed a postal follow‐up questionnaire 3–5 years post‐randomization, which included additional data on alcohol intake, smoking and hysterectomy. Data on hysterectomy were also obtained from Hospital Episodes Statistics (HES England) by identifying all women with Q07 (abdominal excision of the uterus) and Q08 (vaginal excision of the uterus) records and from the ultrasound scan form, which also recorded current HRT use.

### Statistical analysis

Of nearly 270 000 ET measurements, 10 values > 50 mm (maximum of 503 mm) were excluded arbitrarily from the analysis. The remaining ET measurements were transformed to the binary logarithmic scale; the calculated hazard ratios (HR) therefore reflect a doubling of ET.

The association between ET and each cancer type was explored using joint models, that modeled simultaneously the two related processes of serial measurements over time (longitudinal submodel) and time to clinical outcome (survival submodel). Modeling either process in isolation may introduce bias^27^ due to the endogenous nature of the biomarker. Furthermore, joint models use best unbiased linear predictions of the underlying biomarker process in the survival submodel, smoothing out some of the potential measurement error. This can increase the signal and further reduce bias (Appendix S1).

For the longitudinal submodel, current use of HRT at baseline and at each ET measurement, age at LMP, body mass index (BMI), parity and OCP use were included as fixed effects, in addition to a subject‐specific random intercept. The survival submodel included the same adjustment covariates, as well as the association parameter that links the two submodels. We specified the current value link, which is the fitted value including random effects from the longitudinal submodel. The baseline hazard was modeled using a Weibull distribution for all cancers except breast cancer, which used a cubic spline model with one interior knot. Further details of the joint model are given in Appendix S1.

Both longitudinal and survival submodels used age as the time metric, implying delayed entry for the survival analysis. Analysis time was defined as from age at first ET measurement to age at cancer diagnosis of interest. Censorship was age at 31^st^ December 2014^28^, or earlier if there was poststudy‐entry hysterectomy or death. There was no censorship for diagnosis of any other cancer prior to diagnosis of the cancer of interest, nor were they treated as a competing risk. A lung cancer‐specific sensitivity analysis included alcohol and smoking as covariates for both submodels, reducing the sample size by 33%. The joint model failed to converge for the three rarest cancers (NHL, gastric and liver). For comparison, the association between ET and each cancer based on only the baseline measurement was assessed using a Cox model for all nine cancers. The proportional hazards assumption was assessed using the Schoenfeld residuals.

In addition, a standalone longitudinal mixed model, restricted to ET measurements in women with none of the nine investigated cancers or endometrial cancer, was used to construct age‐dependent reference curves at various centiles for normal ET measurements. All statistical analyses were performed using Stata 15.1 (StataCorp. LLC, College Station, TX, USA) and the joint models were fitted using the user‐written command ‘stjm’ ^29^. Hazard functions with 95% CI were estimated for ET values of 2.5 mm and 5 mm (the ratio of which being equivalent to the HR for the log_2_ ET measurements) and presented graphically for the age range, 50–80 years. One‐, 5‐ and 10‐year absolute risk estimates were calculated for selected cancers, ET measurements and ages.

## RESULTS

**Figure 1:**
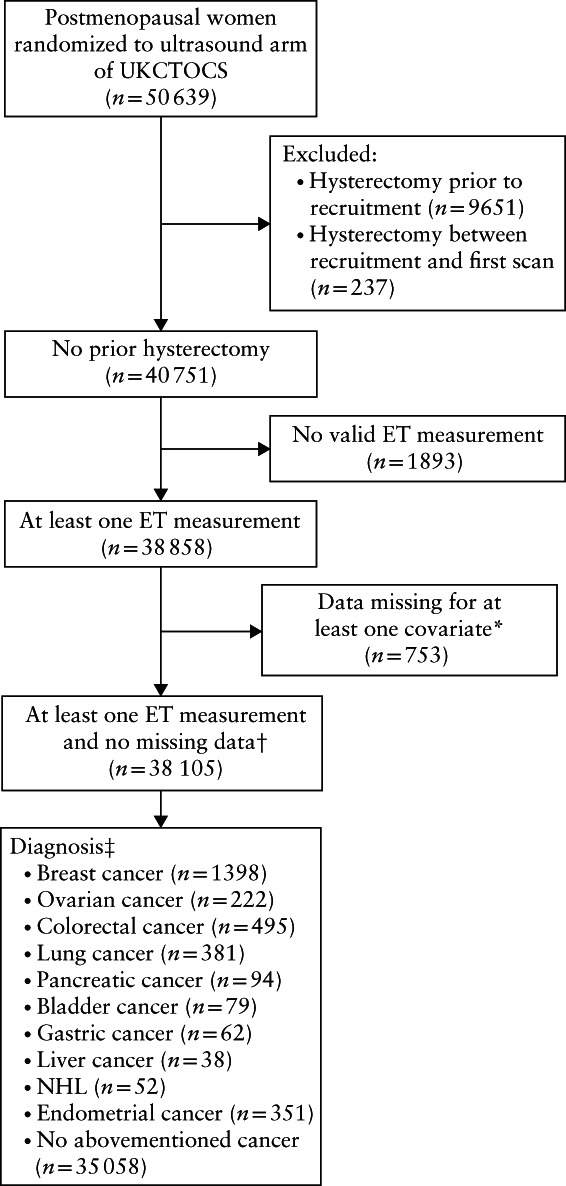
Flow diagram summarizing inclusion of study population. *Body mass index, age at scan, age at menopause, oral contraceptive pill, current hormone‐replacement therapy, parity. †Final cohort. ET, endometrial thickness; UKCTOCS, UK Collaborative Trial of Ovarian Cancer Screening.

Of 50 639 women randomized to the ultrasound arm in UKCTOCS, 9651 had undergone hysterectomy prior to recruitment and 237 underwent hysterectomy between recruitment and the first scan. A further 1893 women had no ET measurements and 753 (1.9%) women had missing data on at least one of the included covariates. The analysis therefore included 38 105 women who had at least one ET measurement and complete data on covariates (Figure 1). Overall, there were 267 567 (36 169 baseline, 231 398 serial) ET measurements, with a median of 8 (interquartile range (IQR), 5–9; maximum, 19) per participant.

At recruitment, the median age of the women was 60.6 (IQR, 56.2–66.2) years, median BMI was 25.6 (IQR, 23.2–28.9) kg/m^2^, median age at LMP was 50.6 (IQR, 47.9–53.1) years and 17.4% of women were currently using HRT. Of the women, 88.0% were parous and 60.4% had used OCP.

**Figure 2:**
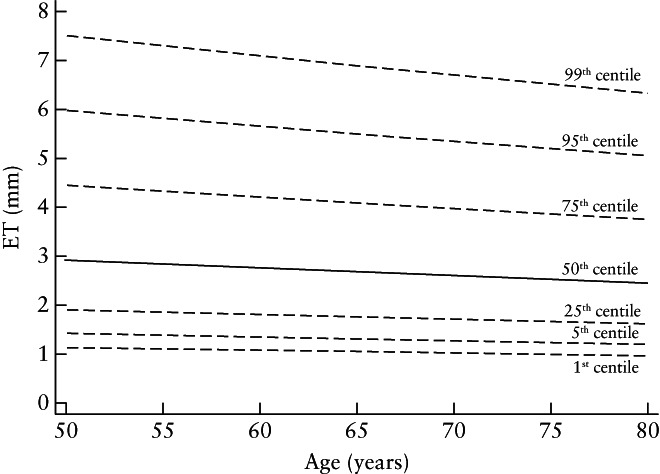
Reference centile curves of endometrial thickness (ET) measured using transvaginal ultrasound in postmenopausal women aged 50–80 years, who were not diagnosed with one of nine investigated or endometrial cancer. Curves are derived from stand‐alone mixed model, which smooths out impact of measurement error at population level.

Table 1 shows the baseline and follow‐up characteristics in the 36 168 women with an ET measurement at baseline, overall and according to baseline ET. Baseline ET was < 3 mm in 18 429 (51.0%) women, ≥ 3 to < 5 mm in 11 694 (32.3%) women and ≥ 5 mm in 6045 (16.7%) women. All factors were strongly associated (*P* < 0.0001) with ET, except for smoking (*P* = 0.254) and OCP use (*P* = 0.021). Age and alcohol use were associated negatively with ET, whereas age at LMP, BMI, parity, HRT use and OCP use were associated positively with ET. Figure 2 depicts the age‐dependent reference curves for estimated ET in women who did not develop any of the described cancers (*n* = 34 922), based on a stand‐alone mixed model. Median ET was estimated to be 2.91 mm in women at age 50 years, with the endometrium thinning slightly over time to 2.46 mm at age 80.

**Table 1:** Baseline and follow‐up characteristics of cohort of 36 168 women undergoing annual transvaginal ultrasound examinations, who had endometrial thickness measurement at baseline, overall and according to baseline endometrial thickness

Characteristic	Overall(*n* = 36 168)	Endometrial thickness			
		< 3 mm(*n* = 18 429)	3 mm to < 5 mm (*n* = 11 694)	≥ 5 mm (*n* = 6045)	*P*
Baseline					
Age (y)					< 0.0001
≥ 50 to < 60	16 840	8197 (48.7)	5742 (34.1)	2901 (17.2)	
≥ 60 to < 70	15 321	8110 (52.9)	4731 (30.9)	2480 (16.2)	
≥ 70	4007	2122 (53.0)	1221 (30.5)	664 (16.6)	
Age at menopause (y)					< 0.0001
< 45	3908	2184 (55.9)	1181 (30.2)	543 (13.9)	
≥ 45 to < 55	27 892	14 304 (51.3)	9020 (32.3)	4568 (16.4)	
≥ 55	4368	1941 (44.4)	1493 (34.2)	934 (21.4)	
BMI (kg/m^2^)					< 0.0001
< 25	15 994	8799 (55.0)	4930 (30.8)	2265 (14.2)	
≥ 25 to < 30	13 147	6721 (51.1)	4238 (32.2)	2188 (16.6)	
≥ 30	7027	2909 (41.4)	2526 (36.0)	1592 (22.7)	
Parity					< 0.0001
Nulliparous	4313	2469 (57.2)	1186 (27.5)	658 (15.3)	
Parous	31 855	15 960 (50.1)	10 508 (33)	5387 (16.9)	
OCP use					0.021
Never	14 207	7368 (51.9)	4505 (31.7)	2334 (16.4)	
Current or past	21 961	11 061 (50.4)	7189 (32.7)	3711 (16.9)	
Current HRT use					< 0.0001
No	30 030	16 383 (54.6)	9379 (31.2)	4268 (14.2)	
Yes	6138	2046 (33.3)	2315 (37.7)	1777 (29.0)	
First scan*					
Current HRT use					< 0.0001
No	31 966	17 126 (53.6)	10 128 (31.7)	4712 (14.7)	
Yes	4202	1303 (31.0)	1566 (37.3)	1333 (31.7)	
Follow‐up questionnaire†					
Smoker					0.254
Never	16 018	8306 (51.9)	5066 (31.6)	2646 (16.5)	
Ever	8395	4266 (50.8)	2735 (32.6)	1394 (16.6)	
Alcohol (units/week)					< 0.0001
None	6384	3275 (51.3)	1948 (30.5)	1161 (18.2)	
≤ 3	10 566	5430 (51.4)	3430 (32.5)	1706 (16.1)	
4–10	7701	4053 (52.6)	2490 (32.3)	1158 (15.0)	
≥ 11	3423	1762 (51.5)	1089 (31.8)	572 (16.7)	

Data are given as *n* or *n* (%).

* Will differ at other scans.

† Approximately 77% completed, 3.5 years post‐randomization.

BMI, body mass index; HRT, hormone‐replacement therapy; OCP, oral contraceptive pill.

During a combined total of 407 838 (median, 10.9; IQR, 9.8–11.8) years of follow‐up, 1398 breast, 381 lung, 495 colorectal, 222 ovarian, 94 pancreatic, 79 bladder, 62 gastric and 38 liver cancers and 52 NHLs were registered. For the joint models, there was a slight difference in sample size between the analyses for each cancer type, which was due to the cancer of interest occurring between recruitment and first ET measurement in some cases. The sample size in the six successfully fitted joint models varied from 38 078 (breast cancer) to 38 105 (ovarian, lung and pancreatic cancer) women (Table 2). In the Cox models, in which only the baseline ET measurement was used due to missing baseline measurements, the sample size was, at most, 1936 lower than that in the joint model. The frequency of cancer was also slightly reduced (Table 2).

**Table 2:** Associations between serial and baseline measurements of endometrial thickness (ET) on transvaginal ultrasound and different non‐endometrial cancers

Cancer	Serial ET measurements*				Baseline ET measurement†			
	Total women (*n*)	Cancer (*n*)	HR (95% CI)	*P*	Total women (*n*)	Cancer (*n*)	HR (95% CI)	*P*
Breast	38 078	1398	1.213 (1.085–1.357)	0.001	36 152	1338	1.063 (0.997–1.133)	0.064
Ovarian	38 105	222	1.390 (1.059–1.824)	0.018	36 169	211	1.215 (1.039–1.420)	0.015
Colorectal	38 099	495	1.147 (0.952–1.381)	0.150	36 164	464	1.037 (0.932–1.155)	0.503
Lung	38 105	381	1.251 (1.015–1.543)	0.036	36 169	364	1.029 (0.911–1.163)	0.645
Pancreatic	38 105	94	0.985 (0.640–1.518)	0.947	36 169	85	0.959 (0.746–1.232)	0.742
Bladder	38 104	79	0.858 (0.524–1.404)	0.542	36 169	76	0.877 (0.668–1.151)	0.345
Gastric	38 104	62	—‡		36 168	59	0.789 (0.579–1.076)	0.135
Liver	38 105	38	—‡		36 169	33	0.858 (0.567–1.299)	0.468
NHL	38 104	52	—‡		36 168	50	0.810 (0.579–1.133)	0.218

Hazard ratios (HR) represent doubling of ET.

All models adjusted by current hormone‐replacement therapy use, body mass index, age at last period, parity and oral contraceptive pill use.

* Joint models included longitudinal and time‐to‐event data.

† Cox models included baseline measurements only.

‡ Joint models did not converge for gastric and liver cancer and non‐Hodgkin's lymphoma (NHL).

In the joint models, there was statistical evidence at the 5% level of a positive association between ET and the risk of three of the six cancer types (Table 2). The strongest association was with breast cancer (HR, 1.21; 95%CI, 1.09–1.36; *P* = 0.001). This model was the only instance in which a Weibull baseline hazard was not suitable (*P* = 0.0002). The one‐year absolute risk of breast cancer for a woman aged 50 with an ET of 5 mm was 165 in 100 000 *vs* 136 in 100 000 for an ET of 2.5 mm. For ovarian cancer, the HR was 1.39 (95%CI, 1.06–1.82; *P* = 0.018) and for lung cancer the HR was 1.25 (95%CI, 1.02–1.54; *P* = 0.036). A sensitivity analysis specifically for lung cancer, that additionally included alcohol intake and smoking as covariates, yielded a higher HR (1.35; 95% CI, 0.98–1.86), although it was not statistically significant (*P* = 0.066) due to fewer events (157/25469). The association between ET and cancer risk was not significant for colorectal (HR, 1.15; *P* = 0.150), pancreatic (HR, 0.99; *P* = 0.947) or bladder (HR, 0.86; *P* = 0.542) cancer. One‐, 5‐ and 10‐year absolute risks for breast, ovarian and lung cancer at selected ages and ETs are shown in Table 3. For example, the 10‐year risk in women at age 60 with an ET of 10 mm is 4.5% for breast cancer, 1.2% for ovarian cancer and 0.9% for lung cancer.

**Table 3:** One, 5‐ and 10‐year absolute risks (AR) for breast, ovarian and lung cancer, according to endometrial thickness (ET) and age

Age (years)	1‐year AR at ET			5‐year AR at ET			10‐year AR at ET		
	2.5 mm	5 mm	10 mm	2.5 mm	5 mm	10 mm	2.5 mm	5 mm	10 mm
Breast cancer									
50	136	165	201	832	1008	1222	2051	2483	3005
55	215	261	317	1229	1490	1805	2750	3327	4022
60	291	353	428	1540	1865	2258	3112	3763	4548
65	327	396	480	1597	1934	2342	3055	3694	4465
70	309	375	454	1481	1795	2173	2827	3420	4134
Ovarian cancer									
50	47	65	90	243	337	468	510	708	982
55	52	72	100	268	372	517	560	778	1079
60	57	79	109	293	407	566	611	848	1177
65	62	86	119	319	443	615	662	919	1275
70	67	93	129	344	478	664	713	990	1373
Lung cancer									
50	15	19	24	94	118	147	242	303	379
55	25	31	39	148	185	232	373	466	583
60	38	48	60	225	281	352	554	693	866
65	57	71	89	330	413	516	800	1000	1249
70	82	102	128	471	589	737	1126	1407	1757

Data are given as risk per 100 000 women.

Table 2 also shows the results of the Cox models for all nine cancers, using only the baseline ET measurement. A significant association at the 5% level was noted only for ovarian cancer (HR, 1.22; 95%CI, 1.04–1.42; *P* = 0.015). HR was < 1 for each of the three cancers not fitted by a joint model (gastric: HR, 0.79; liver: HR, 0.86 and NHL: HR, 0.81), but with *P*‐values > 0.1.

**Figure 3:**
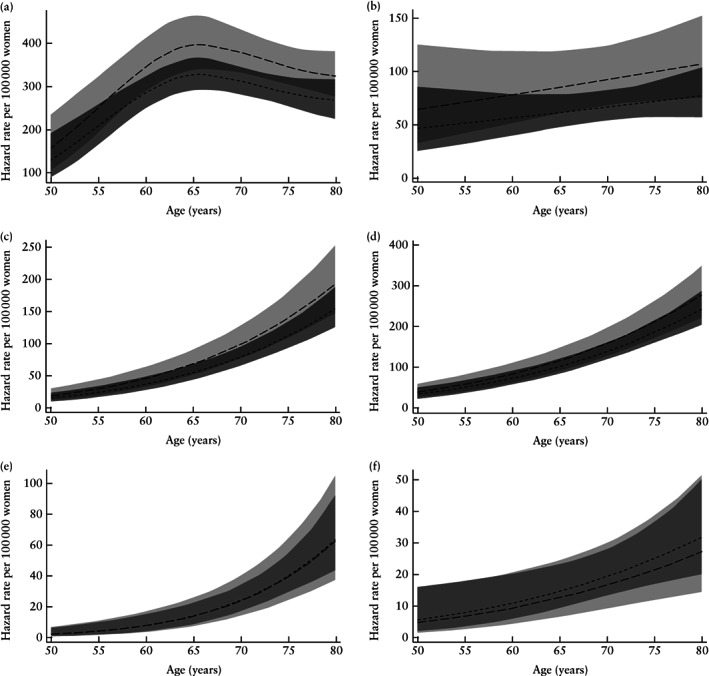
Estimated hazard functions for breast (a; hazard ratio (HR), 1.213; 95% CI, 1.085–1.357), ovarian (b; HR, 1.390; 95% CI, 1.059–1.824), lung (c; HR, 1.251, 95% CI, 1.015–1.543), colorectal (d; HR, 1.147; 95% CI, 0.952–1.381), pancreatic (e; HR, 0.985, 95% CI, 0.640–1.518) and bladder (f; HR, 0.858; 95% CI, 0.524–1.404) cancer, using joint models in women aged 50–80 years, calculated for endometrial thickness (ET) of 5 mm (long dashed line, with shaded 95% CI) and 2.5 mm (short dashed line, with shaded 95% CI); meaning ratio (doubling of ET) equates to model HR. Both functions are estimated for no current hormone‐replacement therapy use, parity of 1, no oral contraceptive pill use, body mass index of 25kg/m^2^ and age at last menstrual period of 50 years and presented per 100 000 women.

Figure 3 shows the estimated hazard functions for the six cancers from the joint models, calculated for ET values of 2.5 mm and 5 mm. Note that overlap of the 95% CI should not be used as an indicator of the significance of the HR values, as the respective hazard functions are not independent. The hazard functions for most of the cancers show increasing risk with age, although the Weibull distribution forces the functions to be monotonic. The exception is breast cancer, for which, after about age 65, there is a gradual decline in the risk associated with ET.

## DISCUSSION

In this prospective cohort study of 38 105 postmenopausal women with a combined total of 267 567 ET measurements, and over 400 000 years of follow‐up, we report on the association between ET measured using TVS, as a functional surrogate for cumulative circulating estrogen, and the risk of nine non‐endometrial cancers that may be affected by estrogen exposure. A doubling of ET was associated with an increased risk of breast (21%), ovarian (39%) and lung (25%) cancer. There was no association with pancreatic cancer, a non‐significant increased risk of colorectal cancer and a non‐significant decreased risk of bladder, liver and gastric cancers and NHL.

Key strengths of this study include its prospective multicenter design, the large cohort size, measurement of ET using a standard protocol and similar ultrasound machines across the centers and over time, completeness of cancer notification through linkage to national cancer registries and long‐term follow up of the cohort. We report for the first time on the association between ET and eight (ovarian, lung, colorectal, pancreatic, bladder, gastric, liver and NHL) cancers. The only prior study investigating the association between ET and breast cancer^24^ either used only the baseline ET measurement or incorporated serial measurements inappropriately. We used joint models, which we believe are an improvement over stand‐alone survival models, including time‐varying Cox models. An additional strength is the development of age‐related normal range reference curves for ET measurements (Figure 2), which may be helpful in a clinical setting.

There is inherent intrasubject variability in ET over time, which may have obscured the association with cancer risk, particularly in a baseline‐only model. Other limitations include the lack of data on histological subtype, which may have indicated stronger associations for those deemed to be estrogen‐dependent. Similarly, we did not have data on HRT type, nor potential confounders such as exercise and diet.

The validity of ET measurement as a surrogate for estrogen exposure has been a topic of debate, with Sit *et al*.^30^ also suggesting that ET measurement is partially valid, as they too noted the difficulty of differentiating endometrial thickening from polyps or fluid in the cavity. Despite this limitation, a potential benefit of using ET as a proxy is that the thickness of the lining should exhibit less day‐to‐day biological variability than circulating estrogen. Furthermore, ET likely reflects the cumulative estrogen exposure, and hence, is a better measure of cancer risk than is estradiol (E2) level.

A doubling of ET resulted in a HR of 1.21 for breast cancer, compared with the HR of 2.00 reported by Felix *et al*.^24^, when comparing the risk in women with ET ≥ 5 mm *vs* < 3 mm. However, the latter report was based on 91 breast cancers compared with 1398 in this analysis, which therefore provides more precise estimates and much greater statistical association. Breast cancer was the only analysis for which the estimated hazard function did not rise monotonically with age, and use of a cubic spline demonstrated a notable decline in the risk associated with increasing ET in women over 65 years of age. This may reflect the age range (50–70 years) of the NHS breast‐screening program and associated overdiagnosis in women of these ages. In postmenopausal women, an increase in breast cancer risk has been reported previously in women with high levels of endogenous sex hormones, including E2 and estrone, with odds ratios of 2.00 and 2.19, respectively, when the highest quintiles are compared to the lowest^3^.

We found a strong association between a doubling of ET and ovarian cancer (HR, 1.39; *P* = 0.018). Trabert *et al*.^31^ reported a modest association of higher levels of estrone and other estrogen metabolites with ovarian cancer, which was limited to non‐serous histotypes. The link between ovarian cancer and exogenous hormones is now well established, with data from the Women's Health Initiative (WHI) suggesting that estrogen‐plus‐progestin therapy may increase ovarian cancer risk, most notably for serous histotypes (relative risk, 1.53)^6^.

The other statistically significant finding was for lung cancer, for which doubling of ET was associated with a 25% increased risk, which would not have been found using the baseline ET value alone (HR, 1.03; *P* = 0.65). This increased risk persisted even after inclusion of the additional covariates of alcohol and smoking, which were available for only a subset of women. This is in keeping with the latest belief that estrogen is responsible for augmenting the risk of lung cancer in smokers^9^, ^12^. One suggestion is that circulating estrogen plays a role in modifying ER‐beta levels, which are known to inhibit tumor growth^10^.

Colorectal cancer was the only other common cancer in our study (*n* = 495), and despite an increase in risk (HR, 1.15), the association with a doubling of ET was not significant at the 5% level (*P* = 0.15). Although increasing circulating estrone has been reported to be associated with an elevated risk of colorectal cancer^15^, the majority of publications suggest that estrogen and its ER‐beta receptor are protective for this cancer^16^, ^17^. Data from the WHI study^32^ and population‐based registries in Norway^33^ have demonstrated a decrease in colorectal cancer risk with short term/current use of estrogen‐plus‐progestin HRT.

Investigation of the five other assessed cancers was limited by the low incidence rates, although the association was estimated to be essentially null only for pancreatic cancer. For bladder (either model), gastric and liver cancer and NHL, the HRs were around 0.85 or lower and it is conceivable that a significant association might be found with greater numbers. These indications of protective effects are broadly consistent with findings from the limited extant studies^18^, ^19^, ^20^.

One of the benefits of using ET as a proxy/surrogate marker for circulating estrogen is its relative ease of measurement. Serum E2 levels are usually measured using a radioimmunoassay. Mass spectrometry is used increasingly because it provides greater specificity and sensitivity, especially for the low E2 concentrations observed in postmenopausal women^34^. However, in addition to cost and run time, there are limited data available on the discrepancies between individual spectrometry‐based assays. Findings from one study indicated that interfering compounds might cause E2 levels to be 10‐times higher than the true value^35^.

In conclusion, in this study exploring the association between ET measured using TVS and the risk of nine non‐endometrial, potentially hormone‐sensitive, cancers in postmenopausal women, we found that high/increasing ET was associated significantly with an increased risk of breast, ovarian and lung cancer. This suggests that ET may merit inclusion in future risk prediction models for these cancers. While our findings need further validation, clinicians might wish to assess appropriately women with high or increasing ET measurements on TVS who do not have endometrial cancer.

## Disclosure

U.M. has stocks in Abcodia Ltd., awarded to her by University College London. I.J. is a coinventor of the Risk of Ovarian Cancer Algorithm (ROCA), which has been licensed to Abcodia Ltd. by Massachusetts General Hospital (MGH) and Queen Mary University of London (QMUL). I.J. has a financial interest in Abcodia Ltd., as a shareholder and director, and is entitled to royalty payments via MGH and QMUL from any commercial use of the ROCA.

## Supporting information


**Appendix** **S1** Supplementary methods
