# Supplementary material for: Serial endometrial thickness and risk of non‐endometrial hormone‐dependent cancers in postmenopausal women in UK Collaborative Trial of Ovarian Cancer Screening
Source: Ultrasound Obstet Gynecol. 2020 Aug 1;56(2):267–75. doi: 10.1002/uog.21894 (PMC7496247; doi:10.1002/uog.21894)
Supplement: Supplementary file 1 — Appendix S1 Supplementary methods [file UOG-56-267-s001.docx]

**Appendix S1** Supplementary methods

Typically, the trajectories of serial data are modelled by longitudinal mixed models that include both fixed effects and random effects. Mixed models can accommodate the serially correlated outcomes within a subject by use of variance components that control for unobserved heterogeneity.^1^ Time-to-event data are modelled using survival methods that allow for censorship, either semi-parametrically, like the Cox model, or with a fully parametric model that estimates the baseline hazard function. If, however, for example, higher biomarker values are associated with risk of cancer then patients who withdraw from the study due to cancer diagnosis, will leave a ‘healthier’ population remaining who have (1) more measurements and (2) typically lower values. This ‘informative dropout’ would result in a longitudinal mixed model with a biased estimate of the mean trajectory, although within UKCTOCS this phenomenon is likely to apply to certain cancers more than others. Strictly though, all biomarkers are endogenous and therefore all serial measurements when related to survival data should use a model that respects the co-dependence.^2^

For cancers other than ovarian, it may seem less conceivable that diagnosis actually results in trial withdrawal, but the formal definition of covariate exogeneity states that the (potential) future path of the biomarker is not affected by a prior failure.^2^ This could only be the case if there was not actually an association at all between biomarker and cancer. Furthermore, an exogenous covariate value should be predictable in the instantaneous moment before time *t*, which is why measurement error itself can be a biasing issue. Finally, a standard time-varying model carries forward the last known measurement in an unsuitable step-function manner, which again introduces bias.^3^

**The Joint Model**

A joint model simultaneously fits a longitudinal sub-model to the serial biomarker data and a survival sub-model to the time-to-event data. The assumption is that the data from both sub-models are co-dependent, and that modelling the data in a joint manner will avoid the biased estimates that may arise if the models were fitted in isolation. This dependence is acknowledged by the inclusion of the ‘association’ parameter in the survival sub-model, representing shared random effects. Instead of using the actual biomarker value in the survival model, the researcher is able to specify a more suitable representation of the data. We used the ‘current value’, which is in fact a fitted value from a longitudinal model, incorporating the best linear unbiased predictions (BLUPs) of the random effects. This could be viewed as an estimate of the underlying biomarker value, if a realistic function for the underlying trajectory is chosen: by including random effects to represent patient-specific unobserved variability in a mixed model, the error term can be apportioned as measurement error. Other choices for the association parameter based on the mixed model predictions, could instead be the first derivative (slope gradient) at each measurement or the value of the random intercept estimate if these were deemed more clinically informative. A joint model can help to produce unbiased results but also be more statistically efficient.^4^ One could legitimately use just the baseline biomarker value in a standard survival model, but this wastes much potential information, and there may be a large time gap between baseline and the event.

A simple two stage model mentioned in the methods involves fitting the longitudinal first, calculating the fitted values at each measurement time-point and then using these as a time-varying covariate in a standard survival model. This approach smooths out the measurement error typical of biomarkers, but like simply using the actual biomarker value implies that the values do not change between observations – visually represented by a step function, rather than a more realistic smoothed function. This approach also does not account for the uncertainty in the predictions carried from the 1^st^ stage to the 2^nd^, and assumes they are fixed quantities, resulting in standard errors that are overly precise. Finally, the two-stage model will still suffer from some of the potential biases of endogenous biomarker values because it fails to consider the data jointly. A joint model will maximise a likelihood function that is the product of both sub-models, so that the solution will respect the fact that a cancer event may have prevented continued observance of biomarker values (informative dropout) or, that at the time of the event, the true biomarker value will likely be quite different to the last recorded value, even if minimal measurement error is present.

Due to the computational burden of modelling over 250 000 ET measurements and 38 000 survival times in a joint manner, and the difficulty of obtaining convergence, endometrial thickness over time was modelled with a random intercept but no random slope, although the mean trajectory allowed fractional polynomials. Ever-use of HRT at baseline, and current-use of HRT at each ET measurement, age at LMP, BMI, parity, and OCP use were included as fixed effects. The survival sub-model included the same adjustment covariates as well as the association parameter. The baseline hazard was modelled using a Weibull distribution for all cancers except breast cancer, where there was evidence from an initial two-stage model fitting process (see above) that a Weibull hazard was unsuitable. Instead, a cubic spline model with one interior knot was judged adequate. By the same two-stage method we also initially assessed the suitability of modelling log_2_ ET, BMI and age at LMP linearly and, similarly, the proportional hazards assumption. Only for endometrial cancer did there appear to be any evidence of non-proportionality. It was not possible to formally model the effect of ET as a time-varying coefficient (TVC) in a joint model setting, so this aspect was explored informally using the results from the two-stage process.

In our joint model we observe for person *i* a continuous longitudinal biomarker $y_{i}$ at time *j* represented by the longitudinal sub-model:

$$y_{i}\left( t_{ij} \right)=m_{i}\left( t_{ij} \right)+ e_{ij} , e_{ij}\sim N(0, \sigma^{2})$$

Where $m_{i}\left( t_{ij} \right)$ is the trajectory function and may be considered the true underlying value of the biomarker for person *i* at time *j*.

The trajectory function is described by an overall mean function (which could include fractional polynomials in the design matrix *X*), and person specific random effects $b_{i}$, as well as additional covariates $w_{i}$ :

$$m_{i}\left( t_{ij} \right)=x_{i}^{'}\left( t_{ij} \right)\beta+z_{i}^{'}\left( t_{ij} \right)b_{i}+w_{i}^{'}\delta, b_{i}\sim N(0, \Sigma)$$

Let $M_{i}(t)=\{m_{i} (s), 0 \leq s\leq t\}$ be the true unobserved longitudinal profile up to time *t*, then a proportional hazards survival sub-model may be described:

$$h(t|M_{i}(t), v_{i} ) = h_{0}(t)exp\{ \varphi^{'}v_{i}+\alpha m_{i}\left( t \right)\}$$

where $h_{0}\left( t \right)$ is the baseline hazard, modelled parametrically, and $v_{i}$ is a vector of baseline time-independent covariates with respective hazard ratios $\varphi$. In our ‘current value’ parameterisation, $\alpha m_{i}$ reflects how the biomarker enters the survival sub-model. As described, $m_{i}$ is the true value estimate at time *t* and $\alpha$therefore is the hazard ratio pertaining to the biomarker – our quantity of interest.

**References**

1. Rabe-Hesketh S, Skrondal A. Multilevel and longitudinal modeling using Stata. 3rd ed. College Station, Tex.: Stata Press Publication; 2012.

2. Rizopoulos D. Joint models for longitudinal and time-to-event data : with applications in R. Boca Raton: CRC Press; 2012.

3. Prentice RL. Covariate Measurement Errors and Parameter-Estimation in a Failure Time Regression-Model. *Biometrika* 1982; **69**(2): 331-42.

4. Gould AL, Boye ME, Crowther MJ, et al. Joint modeling of survival and longitudinal non-survival data: current methods and issues. Report of the DIA Bayesian joint modeling working group. *Statistics in Medicine* 2015; **34**(14): 2181-95.
